# Mitochondria and Calcium Regulation as Basis of Neurodegeneration Associated With Aging

**DOI:** 10.3389/fnins.2018.00470

**Published:** 2018-07-13

**Authors:** Marioly Müller, Ulises Ahumada-Castro, Mario Sanhueza, Christian Gonzalez-Billault, Felipe A. Court, César Cárdenas

**Affiliations:** ^1^Geroscience Center for Brain Health and Metabolism, Santiago, Chile; ^2^Department of Medical Technology, Faculty of Medicine, Universidad de Chile, Santiago, Chile; ^3^Center for Integrative Biology, Faculty of Sciences, Universidad Mayor, Santiago, Chile; ^4^Department of Biology, Faculty of Sciences, Universidad de Chile, Santiago, Chile; ^5^The Buck Institute for Research on Aging, Novato, CA, United States; ^6^Anatomy and Developmental Biology Program, Institute of Biomedical Sciences, University of Chile, Santiago, Chile; ^7^Department of Chemistry and Biochemistry, University of California, Santa Barbara, Santa Barbara, CA, United States

**Keywords:** mitochondria, MAMS, calcium, neurodegeneration, ROS, MPTP, aging, endoplasmic reticulum

## Abstract

Age is the main risk factor for the onset of neurodegenerative diseases. A decline of mitochondrial function has been observed in several age-dependent neurodegenerative diseases and may be a major contributing factor in their progression. Recent findings have shown that mitochondrial fitness is tightly regulated by Ca^2+^ signals, which are altered long before the onset of measurable histopathology hallmarks or cognitive deficits in several neurodegenerative diseases including Alzheimer’s disease (AD), the most frequent cause of dementia. The transfer of Ca^2+^ from the endoplasmic reticulum (ER) to the mitochondria, facilitated by the presence of mitochondria-associated membranes (MAMs), is essential for several physiological mitochondrial functions such as respiration. Ca^2+^ transfer to mitochondria must be finely regulated because excess Ca^2+^ will disturb oxidative phosphorylation (OXPHOS), thereby increasing the generation of reactive oxygen species (ROS) that leads to cellular damage observed in both aging and neurodegenerative diseases. In addition, excess Ca^2+^ and ROS trigger the opening of the mitochondrial transition pore mPTP, leading to loss of mitochondrial function and cell death. mPTP opening probably increases with age and its activity has been associated with several neurodegenerative diseases. As Ca^2+^ seems to be the initiator of the mitochondrial failure that contributes to the synaptic deficit observed during aging and neurodegeneration, in this review, we aim to look at current evidence for mitochondrial dysfunction caused by Ca^2+^ miscommunication in neuronal models of neurodegenerative disorders related to aging, with special emphasis on AD.

## Introduction

In the last century, the population aged over 60 years old has rapidly increased around the world (Beard et al., 2016). Aging is the major risk factor for many chronic diseases such as cancer, diabetes, hypertension, and neurodegenerative disorders (Kennedy et al., 2014). In particular, aging has been correlated with the occurrence of several types of dementia, affecting 5–10% of people over 65, and about 50% of people over 85 years old according to the Alzheimer’s Disease (AD) International (Prince et al., 2015). AD, the most common and still incurable form of dementia, shares several similar cellular alterations with brain aging including mitochondrial dysfunction, oxidative stress, Ca^2+^ dysregulation, and impaired proteostasis (Leuner et al., 2007; Kern and Behl, 2009; Rodrigue et al., 2009; Martinez et al., 2017). Most cases of AD are sporadic (SAD) and characterized by a late onset of symptoms, such as a decline of intellectual and cognitive functions and irreversible memory loss as major features. Several genes have been found to increase the risk of SAD, with the gene for apolipoprotein E (APOE) being the most studied, specifically, the polymorphism that produces the 𝜀4 allele of the APOE, APOE4 variant of the protein (Allen et al., 2012). In addition, nearly 1% of the cases of AD that are dominantly inherited present an early development known as familial AD (FAD) characterized by mutations in presenilin-1 (PS1) and -2 (PS2) or in the amyloid precursor protein (APP; Sherrington et al., 1995). Both SAD and FAD are characterized by neuronal cell death and assumed to be similar to some extent (Hardy and Selkoe, 2002), but the key events prior to cell death are still unclear.

Mitochondria are central organelles in neuronal physiology integrating several crucial functions such as cell respiration, energy metabolism, Ca^2+^ homeostasis, and reactive oxygen species (ROS) generation, all of which have been found to be dysregulated in aging, AD, and other neurodegenerative disorders such as Parkinson’s disease (PD) and amyotrophic lateral sclerosis (ALS)/frontotemporal dementia (FTD) disease (Winklhofer and Haass, 2010; Schon and Przedborski, 2011; Itoh et al., 2013; Manfredi and Kawamata, 2016). Here we present an overview of selected findings regarding mitochondrial dysfunction in neurodegenerative disease and discuss their potential as therapeutic targets.

## Er-Mitochondria Communication and Ca^2+^ Regulation in Age-Associated Neurodegenerative Diseases

**FIGURE 1:**
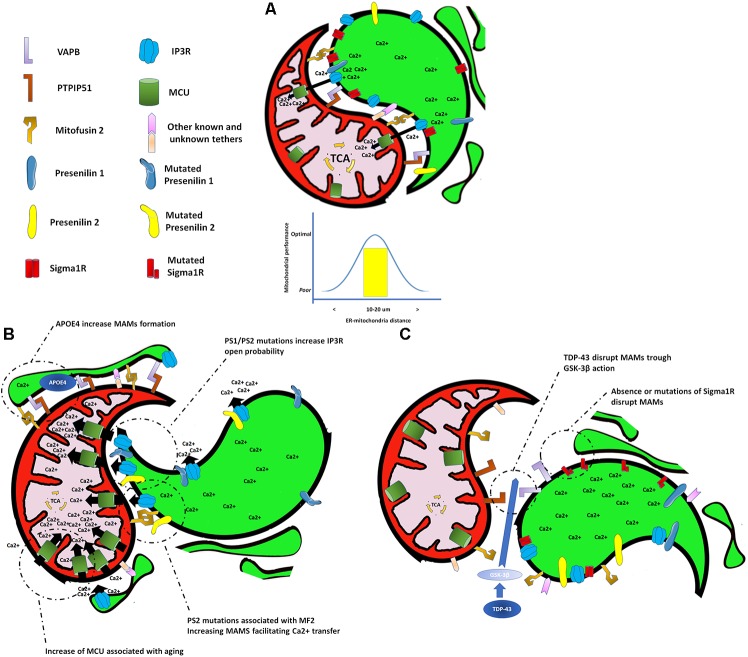
Endoplasmic reticulum and mitochondria interactions under different scenarios. **(A)** Endoplasmic reticulum–mitochondrial interaction at a proper distance allows a correct transfer of calcium to the mitochondrial matrix to sustain bioenergetic function. **(B)** Shortening of the distance at the endoplasmic reticulum–mitochondrial interaction, or an increase in either the mitochondrial calcium uptake or the calcium release from the endoplasmic reticulum, causes an exaggerated transfer of calcium to mitochondria affecting its normal bioenergetic function that leads to cell death. **(C)** Separation of the endoplasmic reticulum and mitochondria causes a decrease in the calcium transfer to the mitochondrial matrix generating a drop in the bioenergetic and metabolic activity of the mitochondria.

Communication between organelles allows cells to function and adapt in a changing cellular environment. The endoplasmic reticulum (ER) and mitochondria couple at specific sites termed mitochondria-associated membranes (MAMs), which integrate and coordinate several cellular functions, including synthesis and exchange of phospholipid, apoptosis, mitochondrial dynamics, and Ca^2+^ signaling (Liu and Zhu, 2017; **Figure 1A**). Remarkably, all these processes are affected early during aging, AD pathogenesis, and other neurodegenerative conditions, suggesting a role for MAMs in the pathogenesis of these diseases (De Vos et al., 2012; Hedskog et al., 2013; Gautier et al., 2016; Area-Gomez et al., 2018). For example, the overexpression of both wild-type and familial ALS/FTD mutant TDP-43 in HEK293, CV-1, and NSC34 cell lines reduces ER–mitochondria associations and Ca^2+^ exchange between these two organelles (Stoica et al., 2014; **Figure 1C**). Likewise, loss of Sigma 1 receptor (which is responsible for some familial forms of ALS/FTD) has been shown to interfere with ER–mitochondria associations (Bernard-Marissal et al., 2015; **Figure 1C**). Conversely, an increase in the lipidic enzymatic function of MAMs and their inter-organelle extension has been described in fibroblasts from patients with SAD, in human SAD brains, and in AD mouse models (Area-Gomez et al., 2012; Hedskog et al., 2013). Remarkably, one of the most common and validated risk factors for SAD, the presence of APOE4 (Holtzman et al., 2012), has recently been associated with an increase in the ER–mitochondrial communication and MAM enzymatic activity (Tambini et al., 2016; **Figure 1B**). Furthermore, MAMs are highly enriched in PS1 and PS2 proteins (Area-Gomez et al., 2009) which when mutated, as in fibroblasts from patients with FAD, also increase the lipidic enzymatic function of the MAMs and ER–mitochondria communication (Area-Gomez et al., 2012), through a mechanism that involves an interaction between the mutated form of PS2 and mitofusin-2 (Mfn2; **Figure 1B**), a key protein in the formation of MAMs (Filadi et al., 2016).

Conditions that increase or decrease the extension of MAMs will affect the transfer of Ca^2+^ from the ER to mitochondria, resulting in either a mitochondrial Ca^2+^ overload, or a lack of Ca^2+^. If the transfer of Ca^2+^ is excessive, cell death occurs (Schinder et al., 1996). If Ca^2+^ transfer to mitochondria is too low, a bioenergetics crisis occurs, also resulting in cell death (Cardenas et al., 2010). Importantly, Ca^2+^ is dysregulated in the aged brain and in AD (Landfield and Pitler, 1984; Gibson and Peterson, 1987; Khachaturian, 1987). Upregulation of Ca^2+^ levels can both initiate and accelerate several AD features, from amyloid deposition to synapse loss (Stutzmann et al., 2007). Several mechanisms have been proposed to explain the upregulation of cytoplasmic Ca^2+^ levels in AD including overexpression of the ryanodine receptor (RyR; Chakroborty et al., 2012), or β-amyloid (Aβ)-triggering release of Ca^2+^ from both extracellular and intracellular sources (Demuro and Parker, 2013). Another mechanism involves an increase of Ca^2+^ leak from the ER through sensitization of the inositol 1,4,5-trisphosphate receptor (InsP_3_R) Ca^2+^ channel by directed interaction with FAD-linked PS mutants (Cheung et al., 2008, 2010) or indirectly by interaction of FAD-linked PS mutants with the SERCA pump (Green et al., 2008). In agreement with the latter, it has been demonstrated that overexpression of the FAD-linked PS2 mutant leads to an increase in the generation of cytosolic Ca^2+^ hot spots, ER–mitochondria tethering, and mitochondrial Ca^2+^ uptake (Zampese et al., 2011). This in turn may result in mitochondrial Ca^2+^ overload and could explain the metabolic dysfunction and cell death observed in AD (**Figure 1B**). On the other hand, decreasing intracellular Ca^2+^ overload, specifically through a reduction of the InsP_3_R protein expression by 50%, normalizes FAD PS-associated Ca^2+^ signaling and rescues the biochemical, electrophysiological, and behavioral phenotypes observed in two different PS1-FAD animal models (Shilling et al., 2014; **Figure 1B**). Altogether, the above findings highlight the importance of MAMs and the transfer of Ca^2+^ from the ER to mitochondria in AD pathogenesis and their potential as a therapeutic target.

The role of MAMs in aging has just begun to be unveiled. Similar to what has been observed in AD models, aging increases the ER to mitochondria Ca^2+^ transfer in long-term culture of hippocampal neurons, with correlates with an increase of the mitochondrial Ca^2+^ uniporter (MCU; Calvo-Rodriguez et al., 2016; **Figure 1B**). Interestingly, a decrease in Ca^2+^ transfer to mitochondria and a dissociation of the MAMs have been described in cardiomyocytes from old mice suggesting that the type of modification that MAMs undergo with aging might be cell specific (Fernandez-Sanz et al., 2014).

## The Mitochondrial Permeability Transition Pore (Mptp) Formation in Age-Associated Neurodegenerative Diseases

Under conditions of Ca^2+^ and/or ROS overload, formation of the mitochondrial permeability transition pore (mPTP) takes place, which corresponds to a non-selective channel formed by a protein complex spanning the outer and inner mitochondrial membranes (Bernardi et al., 2006). In physiological conditions, transient opening of the mPTP can regulate Ca^2+^ levels in the mitochondrial matrix (Ichas et al., 1997). However, dysregulated mPTP opening triggers the release of most matrix metabolites such as ROS, Ca^2+^, and NAD ^+^, leading to loss of the mitochondrial membrane potential, inhibition of oxidative phosphorylation (OXPHOS), and mitochondrial swelling (Elrod and Molkentin, 2013; Rottenberg and Hoek, 2017). Even though several proteins are known to participate in mPTP formation [anion channel VDAC, adenine nucleotide translocator (ANT), mitochondrial ATP synthase (F0F1), phosphate carrier (PiC), and cyclophilin D (CypD; Bernardi et al., 2006; Rao et al., 2014), its detailed structural configuration is not yet entirely known.

The mPTP has been linked to neurodegeneration *in vitro* and *in vivo*. In neural progenitor cells, Aβ-amyloid exposure leads to mPTP opening and a decrease in mitochondrial membrane potential, release of cytochrome C, and cell death (Hou et al., 2014). In human AD brains, Aβ-amyloid binds CypD in mitochondria (Du and Yan, 2010), and CypD deficiency improves mitochondrial function, memory, and learning in an AD mouse model (Du et al., 2008). Aβ-induced neurotoxicity *in vitro* was also attenuated pharmacologically by inhibition of the mPTP using cyclosporine A (CsA) on neural stem cells (Chen et al., 2016). Interestingly, it has been shown that CypD knock-out mice exhibit delayed axonal degeneration, a common feature of diverse neurodegenerative diseases (Barrientos et al., 2011; Catenaccio et al., 2017; Salvadores et al., 2017). Indeed, genetic deletion of CypD delays disease progression in other mouse models of neurodegenerative disorders, including ALS (Martin et al., 2009), PD (Thomas et al., 2012), and multiple sclerosis (Forte et al., 2007). Therefore, novel compounds that inhibit mPTP opening are currently been developed, including sanglifehrin A, N-Me-Ala-6-cyclosporin A, and antmanide (Rao et al., 2014).

Aging also modifies the opening probability of the mPTP (Rottenberg and Hoek, 2017). During aging, the probability of the mPTP opening increases due to higher expression levels of CypD and the CypD-activator p53 (Priami et al., 2015). Furthermore, the expression of HSP90, a chaperone that binds CypD to trigger its degradation, is decreased in aged cells (Lam et al., 2015), which could also increase the mPTP opening probability. This evidence is further supported by a faster Ca^2+^-induced mitochondrial swelling in purified liver mitochondria obtained from aged mice (Goodell and Cortopassi, 1998). Interestingly, CypD is inactivated by the deacetylase SIRT3 (Hafner et al., 2010), a known modulator of longevity in diverse species (Jasper, 2013). The reported decline in SIRT3 activity during aging (Brown et al., 2013) may lead to a greater activation of the mPTP, underscoring the role of mitochondria in longevity and onset of age-dependent neurodegenerative diseases. Interestingly, several modulators of longevity, including metformin, mitochondrial UPR, and caloric restriction inhibit the activation of the mPTP (Bhamra et al., 2008; Altieri, 2013; Amigo et al., 2017), and may contribute to lifespan extension (Rottenberg and Hoek, 2017). A key role for mitochondria in age-related disorders has been associated to broad damaging events including increased ROS production and defects in the regulation of intracellular Ca^2+^ levels, which are directly associated to mPTP activation with profound negative consequences for cell survival. Therefore, mPTP emerges as a potential target for neuroprotection in age-related neurodegenerative conditions.

## Mitochondria, Ros, Aging, and Neurodegeneration

Reactive oxygen species are chemical species that are produced by most cell types. The group of molecules that fulfill the criteria for ROS includes hydrogen peroxide, and the highly reactive species superoxide anion and hydroxyl radical (Wilson et al., 2017). The production of ROS in cells is controlled by enzymatic or non-enzymatic mechanisms. The main source for ROS production in terms of quantitative production is the mitochondria (Holmstrom and Finkel, 2014). Mitochondria produces superoxide anion, a by-product of the inefficient transfer of electrons by the electron transport chain (ETC) during OXPHOS, that is quickly converted into hydrogen peroxide by the action of the superoxide dismutases 1–3 (SOD1–3; Quinlan et al., 2013). Of note, despite mitochondria being the main source of ROS in cells, hydrogen peroxide can be produced by more than 30 different enzymes (Go et al., 2015).

While a huge amount of work in the past focused on the deleterious roles for ROS species in cells and organisms, including the “free radical” or “oxidative stress” theory of aging (Harman, 1956) supported by many studies (Harman, 1992; Cadenas and Davies, 2000; Golden et al., 2002), there is growing evidence in the last decades that ROS may serve physiological functions (Zuo et al., 2015; Sies et al., 2017; Wilson et al., 2017). Related to aging, other studies show that unbalanced ROS production does not modify lifespan in mice under tightly controlled conditions (Van Remmen et al., 2003; Ran et al., 2007). Moreover, it was demonstrated that there is no increased oxidative damage with age (Barja and Herrero, 2000; Kauppila et al., 2017). Currently, it has been proposed that adaptive or hormetic production of ROS is required to maintain several cellular mechanisms including stem cell proliferation and fate determination in the brain (Sena and Chandel, 2012; Chaudhari et al., 2014).

In terms of neurodegeneration associated to aging, it has been reported in AD that the Aβ peptide interacts with the mitochondrial protein termed amyloid binding alcohol dehydrogenase (ABAD) in AD mouse models and in post-mortem samples derived from AD human patients. The functional consequence of such an interaction is an increase in ROS production due to abnormal mitochondrial membrane permeability (Lustbader et al., 2004). Altered OXPHOS increases the generation of ROS (Koopman et al., 2013) and is indeed a hallmark for early AD abnormalities in humans. In fact, samples from human subjects show that mitochondrial-encoded OXPHOS genes are altered in aging, mild cognitive impairment, and AD (Mastroeni et al., 2017). Similarly, AD mouse models have shown that both Aβ and tau protein can induce alterations in mitochondrial proteins involved in OXPHOS (Caspersen et al., 2005; Rhein et al., 2009; Eckert et al., 2010), causing an aberrant ROS generation leading to cellular damage. In addition, ROS are known to cause mitochondrial fragmentation (Wang et al., 2014), which reduces mitochondrial performance (Westermann, 2012) favoring the generation of more ROS and cellular damage associated to it.

## Mitochondrial Dysfunction and Synaptic Deficits

Synapses are neuronal structures in which mitochondria are fundamental (Li et al., 2004) by providing large amounts of ATP required to fuel synaptic vesicle physiology and by acting as a Ca^2+^ buffer modulating cytoplasmic Ca^2+^ signal and hence, neurotransmission (Ghosh and Greenberg, 1995; Verstreken et al., 2005; Gunter and Sheu, 2009; Wan et al., 2012). Synaptic mitochondria are more vulnerable to cumulative damage showing impaired Ca^2+^ uptake capacity and increased propensity to undergo mPTP compared to non-synaptic mitochondria (Scheff et al., 2006). Likewise, in an APP/PS1 AD mouse, synaptic mitochondrial function was significantly more affected than non-synaptic mitochondria (Dragicevic et al., 2010). Synaptic deficit is an early event in the pathogenesis of several neurodegenerative disorders including AD and worsens with disease progression and age (Wilcox et al., 2011). The extent of cognitive decline in AD patients is tightly associated with the extent of synapse loss in specific brain regions including cortex and hippocampus (Scheff et al., 2006; Mattson, 2010). Post-mortem hippocampus from AD patients shows a considerable decrease in dendritic spine density (Ferrer et al., 1990) and transgenic mouse models of AD show age-dependent reduction in spine density before plaque deposition (Lanz et al., 2003). Along these lines, in aged synapses and from AD models, a decline in mitochondrial respiration and signs of mitochondrial damage such as reduced antioxidant contents and increased oxidative stress markers has been described (Du et al., 2010; Quiroz-Baez et al., 2013). Proteomic analysis of aged synaptic mitochondria reveals changes in ETC proteins, antioxidants, and proteins related to mitochondrial dynamics (Stauch et al., 2014). Just recently, through the use of cytoplasmic hybrid (“cybrid”) technology, Yu et al. (2017) were able to recapitulate mitochondrial structural and functional changes observed in AD-affected brains. In this model, their findings demonstrate that AD-affected mitochondria elicited detrimental effects on synaptic development (Yu et al., 2017). How ER vesicles found in the synaptic region and the transfer of Ca^2+^ contribute to the impairment of mitochondria and synaptic formation remains to be explored, but given the dysregulation of Ca^2+^ observed during aging, AD, and other neurodegeneration, an important role is expected. Elucidating the factors that underlie early synaptic dysfunction will be key to prevent the widespread neurodegeneration associated with aging.

## Conclusion

Aging continues to be the most relevant risk factor for AD, the most common form of dementia in the elderly, and other neurodegenerative diseases. Both aging and neurodegeneration are accompanied by a loss in the ability of the cells to adjust and rewire their metabolic networks to keep a tight balance between energy production and expenditure in an ever-changing environment. Mitochondria work as an adaptable metabolic control, a “rheostat,” that integrates inputs from the intra and extracellular environment to generate functional outputs that adjust cell behavior and energy production and consumption. Several lines of evidence suggest that mitochondrial function deteriorates with increasing age and the progression of several neurodegenerative diseases. This supports the notion that aging and the neurodegenerative diseases such as AD may share a common root, the failure of the rheostat program. Since Ca^2+^ is also altered in both conditions and can either energize or overload the rheostat depending on the concentration, understanding how MAM formation is regulated is important. Identifying the players that participate in the regulation to assure a proper Ca^2+^ transfer to mitochondria is critical in order to determine the real potential of this intracellular signaling platform as an intervention candidate to improve aging and hinder the onset of neurodegenerative disease such as AD.

## Author Contributions

MM, UA-C, and CC designed and outlined the structure and contents of the review. MM, UA-C, MS, FC, CG-B, and CC contributed to the literature review, discussion, and writing of the manuscript. All authors contributed equally to the draft revisions and final approval of the version to be published.

## Conflict of Interest Statement

The authors declare that the research was conducted in the absence of any commercial or financial relationships that could be construed as a potential conflict of interest.
